# Targeting histone acetylation to overcome drug resistance in the parasite *Trichomonas vaginalis*

**DOI:** 10.1101/2025.01.07.631743

**Published:** 2025-01-07

**Authors:** Julieta Seifert-Gorzycki, Daniela Muñoz, Ayelen Lizarraga, Lucrecia Iriarte, Verónica Coceres, Pablo H. Strobl-Mazzulla, Natalia de Miguel

**Affiliations:** 1. Laboratorio de Parásitos Anaerobios, Instituto Tecnológico Chascomús (INTECH), CONICET-UNSAM, Buenos Aires, Argentina; 2. Laboratorio de Biología del Desarrollo, Instituto Tecnológico Chascomús (INTECH), CONICET-UNSAM, Chascomús, Argentina; 3. Escuela de Bio y Nanotecnologías (UNSAM), Chascomús, Argentina

## Abstract

Trichomoniasis, caused by the parasite *Trichomonas vaginalis*, is the most common non-viral sexually transmitted infection. Current treatment relies exclusively on 5-nitroimidazole drugs, with metronidazole (MTZ) as the primary option. However, the increasing prevalence of MTZ-resistant strains poses a significant challenge, particularly in the current absence of alternative therapies. Several studies have revealed that the development of metronidazole resistance in *T. vaginalis* is linked to genomic and transcriptional alterations. Given the role of epigenetic regulation in controlling gene expression, we investigated whether targeting histone deacetylase (HDAC) enzymes could influence drug resistance. Treatment of an MTZ-resistant strain (B7268) with the HDAC inhibitor, trichostatin A (TSA), in combination with MTZ enhanced drug sensitivity and induced significant genome-wide transcriptional changes, as revealed by RNA-seq analysis. To identify drug-related genes epigenetically silenced in the resistant strain but highly active in a sensitive strain, we compared the expression levels of the genes affected by TSA and MTZ treatment with their baseline expression profiles in both resistant and sensitive strains. This analysis identified 130 candidate genes differentially expressed in the sensitive strain NYH209, less expressed in the resistant B7268 strain, that exhibited significant expression changes upon TSA and MTZ treatment. Functional validation involved transfecting the B7268 strain with plasmids encoding four individual candidate genes: a thioredoxin reductase (TrxR), a cysteine synthase (CS), and two genes containing Myb domains (Myb5 and Myb6). Overexpression of three of these genes resulted in a marked reduction in MTZ resistance, demonstrating their role in modulating drug sensitivity. Our findings identified three novel genes that modulate drug resistance in *T. vaginalis*. This study reveals a previously unknown epigenetic mechanism underlying drug resistance and highlights the therapeutic potential of targeting epigenetic factors, such as HDACs, to overcome resistance and improve treatment efficacy.

## INTRODUCTION

Eukaryotic microbial pathogens are major contributors to global morbidity and mortality. While drug therapies have proven effective against many infections, the emergence of drug resistance underscores the urgent need for alternative treatments. Protozoan infections, in particular, face growing drug resistance challenges, partly due to unsupervised treatments and the misuse of chemoprophylaxis. Resistance in the anaerobic protozoan *Trichomonas vaginalis* was first reported in 1962 [1] and has continued to rise [2]. This parasite causes trichomoniasis, the most prevalent non-viral sexually transmitted disease, with an estimated 276 million new cases annually [3]. Although infections are generally mild or asymptomatic, disease can manifest vaginitis or urethritis as well as infertility, preterm delivery and premature rupture of membranes if acquired during pregnancy [4]. Furthermore, *T. vaginalis* has also emerged as an important cofactor in amplifying human immunodeficiency virus (HIV) transmission and has been linked to increased risk of malignant cervical and prostate cancers [5–8].

The 5-nitroimidazole drugs, particularly metronidazole (MTZ) and tinidazole, are the only approved treatments for trichomoniasis with metronidazole being the most widely prescribed [9]. MTZ functions as a prodrug, requiring reduction of its nitro group to exert its antimicrobial effects [2]. It damages DNA, forms protein adducts and induces oxidative stress by depleting thiol pools [10]. However, MTZ resistance has emerged, affecting approximately 10% of infections in the U.S [9]. Resistance mechanisms fall into two categories: aerobic resistance, associated with oxygen stress responses, and anaerobic resistance, linked to hydrogenosome metabolism deficiencies [11]. Aerobic resistance involves enhanced activity of oxygen-scavenging enzymes (e.g., superoxide dismutase, flavoprotein oxidases), which protect the parasite from oxidative stress. In this context, oxygen serves as the preferred electron acceptor, preventing MTZ reduction and activation in hydrogenosomes [10, 12, 13]. Anaerobic resistance, on the other hand, arises from downregulation of hydrogenosome enzymes such as pyruvate:ferredoxin oxidoreductase (PFOR) and ferredoxin (Fd) [12–14], critical for MTZ activation. Interestingly, certain proteins, such as flavin reductase 1 (FR1), are implicated in both resistance types, as they are often absent or downregulated in both aerobic and anaerobic metronidazole-resistant isolates [10, 12]. MTZ resistance studies have primarily focused on candidate genes related to oxygen scavenging and hydrogenosome metabolism [12, 15–18]. Despite significant progress, disruptions in known genes associated with MTZ resistance do not fully explain this phenomenon in all isolates. Large-scale genetic analyses have revealed specific single nucleotide polymorphisms associated with resistance [15], while changes in gene expression have also been implicated [17, 19–21]. In agreement, transcriptomic studies in *Entamoeba histolytica* further support the association between MTZ resistance and transcriptional changes [22].

Addressing drug resistance remains a significant therapeutic challenge. Over the past decade, epigenetic strategies have shown promise in overcoming drug resistance in cancer, particularly with epidrugs—compounds targeting epigenetic modulators [23–25]. These agents can reverse resistance phenotypes by reactivating or silencing aberrantly regulated genes [26]. A recent survey of drug resistance in cancer cells showed that an inhibition of histone deacetylases (HDAC) activity could prevent the development of drug resistance [27]. Inhibiting HDAC enzymes promotes accumulation of the acetylated form of histone proteins, ultimately leading to less-condensed packaging of genes in chromatin which may lead to the re-expression of silenced genes. HDAC inhibitors (HDACIs) have great potential for treating cancers, immunological diseases and other conditions [28, 29] as they can block angiogenesis, arrest cell growth and lead to differentiation and apoptosis in tumor cells [30]. While HDACIs are extensively studied in cancer, their role in drug resistance in infectious diseases is less understood. Leveraging insights from cancer, epidrugs may offer novel therapeutic options for parasitic diseases. Previous findings demonstrated that histone acetylation influences both transcription and pathogenesis in *T. vaginalis* [31, 32]. Specifically, treating a poorly adherent strain of *T. vaginalis* with the HDACi Trichostatin A (TSA) increased H3KAc and chromatin accessibility around transcription start sites, upregulating genes involved in adherence [31]. Recognizing the pivotal role of chromatin and histone acetylation in orchestrating drug resistance in cancer cells and considering the influence of histone acetylation on gene regulation in *T. vaginalis*, we hypothesized that HDACs contribute to MTZ resistance in this parasite. Studies in related protozoa, such as *Plasmodium falciparum* and *Trypanosoma* spp., have linked HDAC activity to drug resistance and persistence [33–35]. For example, HDACs in *P. falciparum* mediate silencing of drug transport genes, reducing drug uptake [36] while in *T. brucei*, they regulate telomeric silencing of variant surface glycoprotein (VSG) expression sites, facilitating immune evasion and resistance [37]. HDAC inhibitors, including TSA and suberanilohydroxamic acid (SAHA), have been shown to induce global histone hyperacetylation, reactivating silenced genes and disrupting resistance mechanisms in *P. falciparum* and other protozoa [33, 34, 38].

Our findings reveal a novel epigenetic mechanism involving histone acetylation in modulating MTZ resistance in *T. vaginalis*. Treatment of a *T. vaginalis* strain resistant to MTZ (B7268) with a HDAC inhibitor (trichostatin A) and MTZ led to increased drug sensitivity and significant genome-wide transcriptional alterations analyzed by RNA-seq. To identify drug-related genes epigenetically silenced in the resistant strain but highly active in a sensitive strain, we compared the expression levels of the genes affected by TSA and MTZ treatment with their baseline expression profiles in both resistant and sensitive strains. This analysis identified 130 candidate genes differentially expressed in the sensitive strain NYH209, less expressed in the resistant B7268 strain, that exhibited significant expression changes upon TSA and MTZ treatment and might be involved in regulation of drug resistance. Functional validation of some of these genes involved transfecting the B7268 strain with plasmids encoding four individual candidate genes: a thioredoxin reductase (TrxR), a cysteine synthase (CS), and two containing Myb domains (Myb5 and Myb6). Overexpression of three of these genes resulted in a marked reduction in MTZ resistance. Our findings identified three novel genes that modulate drug resistance in *T. vaginalis*. Understanding these mechanisms reveals a previously unknown epigenetic mechanism underlying drug resistance but also opens new avenues for designing effective antiparasitic therapies.

## MATERIALS AND METHODS

### Parasites, cell cultures, and media

*Trichomonas vaginalis* strains BRIS/92/STDL/B7268 [39, 40], SD7 [41], CDC1132 (MSA1132; Mt. Dora, Fla, USA 2008) [42] and NYH209 (ATCC 50146) [44] were cultured in Tryptose Yeast Maltose (TYM) medium [43] supplemented with 10% fetal equine serum and 10 U/ml Penicillin/Streptomycin. B7268 strain was obtained from a Brisbane patient with refractory infection that displays both high aerobic and anaerobic resistance to metronidazole [40]. All parasite strains were grown at 37°C and passed daily.

### Metronidazole susceptibility assay and trichostatin-A treatment

The MTZ susceptibility of *T. vaginalis* was determined by microscopy using a standard minimum lethal concentration (MLC) protocol under aerobic conditions. 2×10^5^ parasites/ml from the log-phase of growth were exposed to serial dilutions of metronidazole (from 0 to 400 μg/ml) (Sigma-Aldrich). MTZ was dissolved in DMSO for preparation of stock solutions. The control tube (0 μg/mL MTZ) received an equivalent volume of DMSO to match the maximum DMSO volume used in the treatment. The final volume for each MTZ concentration was calculated to ensure the DMSO concentration did not exceed 0.1% (v/v). The tubes were incubated aerobically for 48 h and examined with an inverted microscope at 40x magnification for motile trichomonads. The lowest drug concentration at which no motile parasites were observed was recorded as the MLC. The failure of non-motile trichomonads to proliferate following reinoculation into drug-free medium served as confirmation of the end points. Assay were performed four times. The cell density of B7268, CDC1132 and SD7 parasites previously treated with 100 nM Trichostatin A (TSA) for 16 hours was monitored for 48 hours after treatment with different concentrations of MTZ compared to the cell density of untreated control.

### RNA-seq of *T. vaginalis*

*Trichomonas vaginalis* strain B7268 treated with TSA (100 nM), with metronidazole (MTZ; 5 μg/ml), or with a combination of TSA and MTZ, as well as untreated parasites as control were sequenced in triplicates. Total RNA was extracted from ~5 × 10^6^ *T. vaginalis* according to the protocol outlined in the Total RNA Purification Kit (Norgen Biotek Corp.) following the manufacturer’s instructions. RNA quality and quantity were assessed using an Agilent Bioanalyzer with RNA integrity numbers (RINs) of >7 for all samples. The mRNA libraries were paired-end (100 bp) sequenced with Illumina using TruSeq Stranded mRNA (Macrogen, Inc).

### Transcriptome analyses

After sequencing, 20–26 million reads were generated per RNA-seq library. For quality control of the paired-end sequencing data, the software FastQC [44] was used. The adapter sequence content was identified and trimmed using Trimmomatic [45]. Then, HISAT2 [46] was used to align the RNA-seq data sets to the G3 reference genome sequence, and the results showed an overall alignment rate of >90% for all libraries. In order to quantify the counts read for each gene, featureCounts [47] was used with default parameters. Principal components analysis was carried out to evaluate the variation through biological replicates. Using DESeq2 package, it was possible to identify up- and down-log2 fold changes in gene expression [48]. Differential gene expression and dispersion were examined using MAplot and plotDispEsts functions, respectively. Only changes where |log2FC| > 1 and padj < 0.05 were considered significant. Using the results command from DESeq2, the expression patterns of the three treatments (B7268-MTZ, B7268_TSA, and B7268_TSA+MTZ) were compared pairwise against the control group. In the same way, the comparison between NYH209 vs B7268 was obtained, in order to identify the genes differentially expressed in a susceptible strain to MTZ. NYH209 sequencing dataset were obtained from Sequence Read Archive (SRA) in online repositories (BioProject accession code PRJNA1188544). For the final list of candidates genes a Gene Ontology (GO) enrichment analysis was carried out using the TrichDB database of open access [49]. P-value ≤ 0.05 was considered significantly enriched.

### Parasite viability assays

Parasite viability was analyzed using the fluorescent exclusion dye propidium iodide. For this purpose, wild-type B7268 (control) as well as B7268 parasites treated with 100 nM of TSA for 16 h treated were labeled with propidium iodide (PI, 20 μg/ml) at 4°C for 10 min. PI fluorescence associated with non-viable cells was measured by flow cytometry (at λ = 544/602 nm) on FACSCalibur (Becton–Dickinson) and analyzed using Flowing software version 2.4.1 (Perttu Terho, Turku Centre for Biotechnology, Finland; www.flowingsoftware.com). Four independent experiments were performed.

### Plasmid construction and exogenous expression in *T. vaginalis*

Master-Neo-(HA)_2_ plasmids [50] containing one candidate gene each (TrxR, CS, Myb5, Myb6) were purchased from GeneScript (USA). Electroporation of *T. vaginalis* strain B7268 was carried out as described previously [50] with 50 μg of circular plasmid DNA. Parasites were transfected in parallel with an empty plasmid (EpNEO) to be used as control. Transfectants were selected with 100 μg/ml G418 (Sigma).

### Immunolocalization experiment

Parasites were incubated at 37°C on glass coverslips for 4 h. The parasites were then fixed and permeabilized in cold methanol for 10 min. The cells were washed and blocked with 5% fetal bovine serum (FBS) in phosphate buffered saline (PBS) for 30 min, incubated with a 1:500 dilution of anti-HA primary antibody (Covance, Emeryville, CA, USA) diluted in PBS plus 2%FBS, washed and then incubated with a 1:5000 dilution of Alexa Fluor-conjugated secondary antibody (Molecular Probes). The coverslips were mounted onto microscope slips using ProLong Gold antifade reagent with 4′, 6′-diamidino-2-phenylindole (Invitrogen). Fluorescence parasites were visualized using a Zeiss Axio Observer 7 (Zeiss) microscope.

## RESULTS

### Treatment with the histone deacetylase inhibitor TSA overcome metronidazole drug resistance in *T. vaginalis*

**Figure 1:**
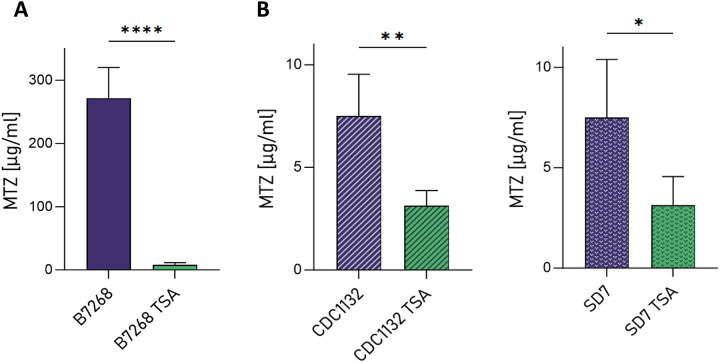
A. B7268 sensibilization to MTZ upon TSA co-treatment. B. The sensibilization to MTZ upon TSA co-treatment was also observed in CDC1132 and SD7 *T. vaginalis* strains. For each strain four independent experiments were conducted. Data are expressed as concentration of MTZ [μg/ml] for which the parasites were no longer viable ± standard deviation of the mean. Student T-tests (α=0.95) were used to determined significant differences between treatments. (Signif. codes: ns = P-value > 0.05, * = P-value ≤ 0.05, ** = P-value ≤ 0.01, *** = P-value ≤ 0.001, **** = P-value ≤ 0.0001)

We hypothesize that administering epigenetic drugs capable of influencing chromatin compaction could restore sensitivity to metronidazole (MTZ) in drug-resistant parasites. This approach may involve reactivating silenced genes implicated in the development of MTZ resistance. To test this hypothesis, we treated a highly resistant *T. vaginalis* strain (B7268) [40] with the histone deacetylase (HDAC) inhibitor Trichostatin A (TSA) and evaluated the resistance levels to MTZ using a standard minimum lethal concentration (MLC) protocol under aerobic conditions. Resistant parasites were pretreated with 100 nM TSA for 16 hours, followed by MTZ treatment for 48 hours. This concentration of TSA has previously been shown to increase H3KAc, relax the chromatin and modulate gene expression in *T. vaginalis* [31]. As shown in Figure 1A, TSA pretreatment effectively restored metronidazole sensitivity in resistant B7268 parasites without compromising survival in MTZ-untreated cells. This sequential treatment with an epigenetic drug sensitized refractory B7268 parasites, overcoming MTZ resistance in *T. vaginalis*. To further validate these findings, we tested the efficacy of TSA and MTZ sequential treatment in drug-sensitive *T. vaginalis* strains SD7 and CDC1132 (Fig. 1B). Although the effect was more pronounced in drug-resistant parasites, TSA treatment also influenced drug sensitivity in the SD7 and CDC1132 strains. These results underscore the potential of epigenetic modulation as a promising strategy to reverse MTZ resistance in *T. vaginalis*. By targeting drug resistance mechanisms through chromatin remodeling, epigenetic drugs may offer a novel therapeutic approach to combatting resistant *T. vaginalis* infections.

### Histone acetylation and transcriptomic insights into MTZ resistance modulation

**Figure 2:**
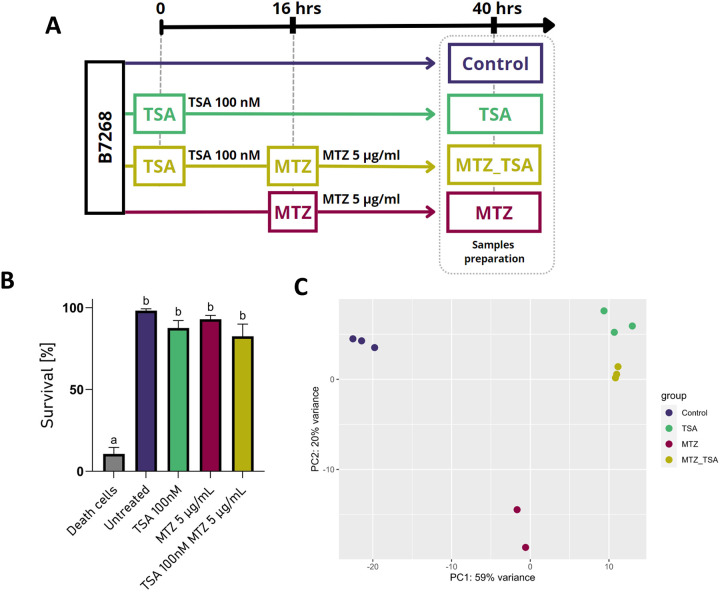
A. RNA-seq experimental Design. The RNA-seq experiment was designed with four treatment groups: (1) untreated B7268 parasites control, (2) B7268 was treated with 100 nM Trichostatin A (TSA), (3) B7268 was treated with 5 μg/ml metronidazole (MTZ) and (4) B7268 was treated with 100 nM TSA for 16 h followed by a treatment w 5 μg/ml metronidazole (TSA+MTZ). All samples, including controls, were prepared for sequencing at the same time, 40 hours after the start of the experiment. B. Viability of B7268 parasites upon treatments with TSA [100 nM], MTZ [5 μg/ml] and TSA [100 nM] MTZ [5 μg/ml] were analyzed by flow cytometry using propidium iodide. Data are expressed as mean values ± standard deviation of four independent experiments. ANOVA followed by Tukey’s post hoc test (α=0.999) was used to determine significant differences. C. Principal components analysis of the four treatment groups and its replicates based on normalized gene read counts. The x-axis represents principal component one (PC1) and explains 59% of the variation, while the y-axis represents principle component two (PC2), and explains 20% of the variation.

**Figure 3:**
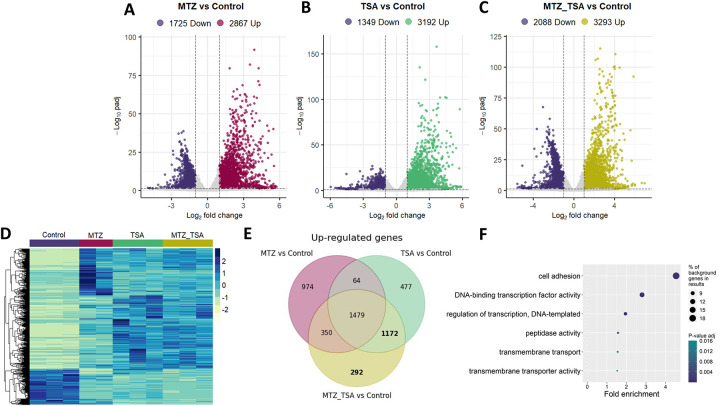
Volcano plot distributions of differentially expressed genes (DEGs) obtained from RNAseq analysis of B7268 treated with MTZ vs Control (A), B7268 treated with TSA vs Control (B) and B7268 treated with MTZ and TSA vs Control (C). The selective condition to determine DEGs was defined as |log2FC| > 1 and p-value adjusted < 0.05. (D) Heatmap displaying normalized expression of DEGs obtained throughout the different treatments. Each horizontal line representing an individual gene. Color gradient represents the expression level. (E) Venn diagram showing the number of up-regulated genes shared by the three comparisons. The genes of interest are highlighted in bold. (F) Gene ontology enrichment analysis of molecular function for genes of interest.

Histone acetylation is a well-documented mechanism that relaxes chromatin structure, enabling transcription factors to enhance gene expression [51]. To examine the molecular basis for the effect of TSA in modulation of MTZ resistance, we conducted whole-transcriptome RNA-seq analysis of the *T. vaginalis* B7268 resistant strain under different treatment conditions: untreated control, TSA (100 nM), MTZ (5 μg/ml), and sequential TSA and MTZ treatment (Fig. 2A). TSA treatment decreased the parasite growth rate without affecting viability (Fig. 2B). Libraries were sequenced in triplicates, and PCA revealed clear separations between treatments, with PC1 and PC2 accounting for 59% and 20% variance, respectively, excluding experimental variations among replicates (Fig. 2C). The transcriptome analysis showed that TSA-treated parasites displayed a closer similarity to sequential TSA and MTZ-treated parasites than to MTZ-treated or untreated controls (Fig. 2C and 3D). To identify those genes whose expression is most strongly associated with changes in MTZ resistance due to TSA treatment, we compared each of the treatments (TSA, MTZ or sequential treatment with TSA and MTZ) with the corresponding gene expression in untreated B7268 parasites (control) (Supplementary Table 1). The results showed marked changes in gene expression (≥ or ≤ 2X) in control cells compared to parasites following treatment with MTZ (Fig 3A), with TSA (Fig 3B) or sequential treatment with TSA and MTZ (MTZ_TSA) (Fig 3C). Specifically, treating B7268 resistant parasites with MTZ showed marked changes in gene expression: 2867 up-regulated and 1725 down-regulated genes upon treatment (Fig 3A). As expected, the number of genes overexpressed upon TSA treatment (3192 genes) is higher than the number of repressed genes (1349) (Fig 3B), indicating that HDAC inhibition acts primarily by promoting the transcription of a set of genes. When parasites are sequentially treated with TSA and MTZ, 3293 upregulated and 2088 downregulated genes were detected when compared to untreated control cells (Fig 3C). The heatmap of differentially expressed genes revealed a distinct gene expression pattern between treated and untreated groups (Fig 3D). When comparing the upregulated genes in each of the treatments (TSA, MTZ or sequential treatment with TSA and MTZ), all three treatments shared 1479 upregulated genes (Fig 3E).

Histone acetylation, a well-established mechanism for relaxing chromatin structure and augmenting gene expression [30]. Considering this, we focused on identifying genes whose expression was modulated by TSA-induced chromatin changes and potentially associated with MTZ resistance. We selected two groups of upregulated genes: (1) genes upregulated in both TSA and TSA+MTZ treatments (1172 genes) and (2) genes exclusively upregulated in TSA+MTZ treatment (292 genes), resulting in 1464 candidate genes (from now named candidate genes) (Fig. 3E). The 1464 candidate genes were manually grouped by their common function to have an overview of which processes are influenced by the presence of TSA and MTZ (Table 1 - Supplementary table 2). Gene Ontology analysis indicate that most significantly enriched terms identified in the displayed genes included those related to cell adhesion, DNA-binding transcription factor activity, and peptidase activity, as well as genes involved in transmembrane transport processes, such as drug transmembrane transporters (Fig. 3F). Previously identified genes involved in MTZ activation, such as thioredoxin reductase (TVAGG3_0315040) and nitroreductase genes (TVAGG3_0444150, and TVAGG3_0873820), were significantly upregulated in TSA+MTZ-treated parasites, supporting their role in MTZ sensitivity (Supplementary Data 2). Furthermore, proteins from the diflavin flavoprotein A 2-related family, flavodoxin-like fold, and flavoprotein family (TVAGG3_0421620, TVAGG3_0583440, TVAGG3_0080430) were also upregulated in MTZ_TSA group. Notably, redox-related proteins like flavodoxins and hydrogenosomal enzymes (e.g., PFOR and Fd) showed varied expression, suggesting a complex interplay in resistance mechanisms. One Fd and three PFOR genes (TVAGG3_0476390, TVAGG3_0282970, TVAGG3_0890230, TVAGG3_0998520) were downregulated in both MTZ_TSA and TSA groups, meaning that the transcription of these genes seems to be not regulated by histone acetylation.

### Differences in gene expression among MTZ-resistant relative to MTZ-sensitive *T. vaginalis* strains

**Figure 4:**
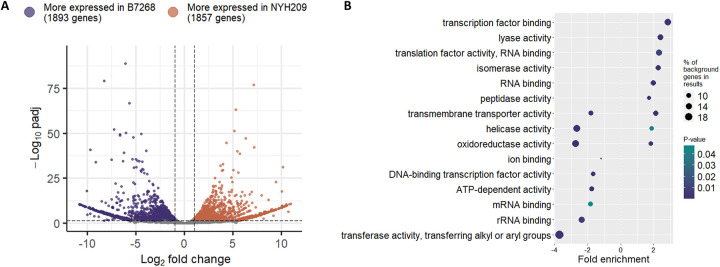
A. Volcano plot distribution of diffrenetially expressed genes (DEGs) obtained from RNAseq analysis of B7268 and NYH209 *T. vaginalis* strains. DEGs was defined as |log2FC| > 1 and p-value adjusted < 0.05. B. Gene ontology enrichment analysis of molecular function for DEGs genes for each strain (left: B7268 and right: NYH209).

To identify transcriptional changes naturally associated with MTZ-resistance, we performed RNA-seq analysis in two different *T. vaginalis* strains: a MTZ resistant (B7268) and a MTZ sensitive (NYH209) strains (Fig. 4). PCA was conducted to examine the relationship between the samples, showing a clear distinction between treatments (PC1, 95% and PC2, 4% variance) and accounting for minimal experimental variation among replicates (Supplementary Fig. 1). Our results detected 3750 differentially expressed genes (DEGs) (adjusted p-values ≤ 0.05) (Fig 4), including 1857 genes more expressed in NYH209 sensitive strain and 1893 genes more expressed in the B7268 resistant strain (Fig 4A; Supplementary Table S3). As we proposed that genes involved in drug resistance might be epigenetically silenced in a resistant strain, we analyzed the genes differentially expressed in the sensitive strain NYH209 compared to the resistant strain B7268. GO analysis of differentially expressed genes demonstrated that genes more expressed in NYH209 are associated with the activation of many different pathways, including cellular and metabolic process, nuclear division, DNA recombination, among others (Fig 4B). In contrast, genes more highly expressed in B7268 were linked to pathways such as sulfur metabolic process, ribosome biogenesis, and tRNA metabolic process (Fig 4B).

**Figure 5:**
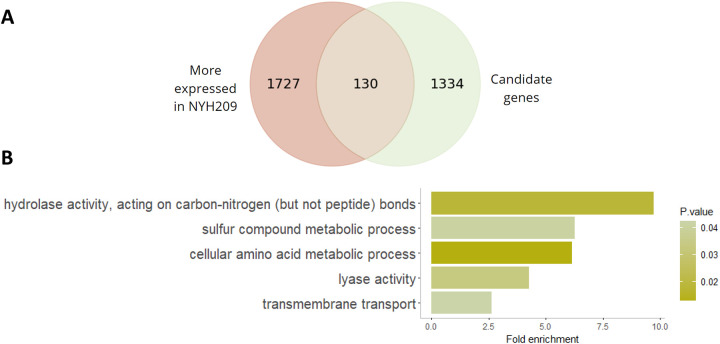
A. Overlap between the more expressed genes in the MTZ-susceptible strain (NYH209) and the 1464 candidates genes plotted as Venn diagram. B Gene ontology enrichment analysis of biological processes for the 130 genes shared by NYH209 and the candidates genes.

By intersecting the 1464 candidate genes that were influenced by TSA and MTZ treatment and the 1857 genes differentially expressed in NYH209 sensitive strain, we identified 130 genes potentially epigenetically silenced in B7268 but activated by TSA treatment (Fig 5A and Supplementary Data 4). These genes, involved in processes like sulfur metabolism, amino acid biosynthesis, and transmembrane transport (Fig 5B), likely contribute to MTZ resistance modulation.

### Overexpression of candidate genes in a highly resistant isolate B7268 significantly decrease MTZ resistance

**Figure 6:**
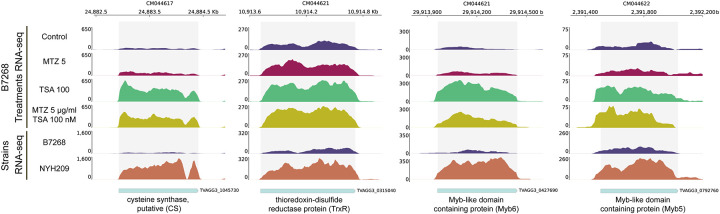
The figure displays the genome browser screenshots of normalized read coverage for six genes: cysteine biosynthetic process (TVAGG3_1045730), protein ubiquitination (TVAGG3_0534480), transcription inititiator DNA-binding domain Idb family (TVAGG3_0649430), thioredoxin-disulfide reductase (TVAGG3_0315040) and two RNA polymerase II transcription regulator recruiting proteins (TVAGG3_0792760 and TVAGG3_0427690). RNA-seq analysis of each gene expression profiles for B7268 strain under different treatments (top) as well as comparison with the correspondent gene expression in a sensitive strain (bottom). Each panel represents a gene locus, with shaded regions indicating annotated coding sequences. Y-axis values correspond to normalized read counts

**Figure 7.**
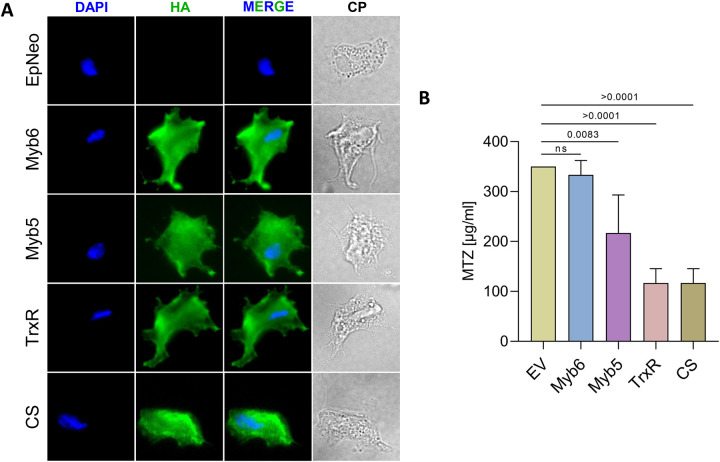
A. Subcellular localization of four selected putative genes involved in modulation of drug resistance: cysteine biosynthetic process (CS, TVAGG3_1045730), thioredoxin-disulfide reductase (TrxR, TVAGG3_0315040) and two RNA polymerase II transcription regulator recruiting proteins (Myb5, TVAGG3_0792760 and Myb6, TVAGG3_0427690). Cells expressing C-terminal HA-tagged versions of the indicated gene were stained for immunofluorescence microscopy using a mouse HA-tagged antibody. The nucleus (blue) was also stained with 4-,6-diamidino-2-phenylindole. B. The level of MTZ resistance of parasites from the B7268 strain transfected with CS, TrxR, Myb5 and Myb6 was assessed and compared with parasites transfected with empty plasmid (EpNEO). Four independent experiments were performed. Data are expressed as -fold increase compared with empty plasmid control ± the standard deviation of the mean.

To validate the involvement of the 130 epigenetically silenced genes in drug resistance modulation in B7268, we selected four candidate genes for functional analysis: cysteine synthase (CS, TVAGG3_1045730), thioredoxin-disulfide reductase (TrxR, TVAGG3_0315040), and two Myb-like domain-containing proteins, Myb5 (TVAGG3_0792760) and Myb6 (TVAGG3_0427690) (Figs. 6 and 7). Transcriptional profiling revealed that these genes exhibited low expression levels in untreated B7268 parasites as well as those treated with MTZ (Fig. 6). However, treatment with TSA or TSA+MTZ significantly upregulated their expression (Fig. 6). In contrast, the naturally sensitive strain NYH209 consistently showed high expression of these genes (Fig. 6), supporting the hypothesis that epigenetic silencing in the resistant strain contributes to reduced MTZ sensitivity.

To test whether exogenous expression of these four candidate genes in the highly resistant B7268 strain would confer an MTZ-sensitive phenotype, the genes were cloned into episomal expression vectors under the control of a strong promoter and transfected independently as C-terminal HA-tagged fusion proteins. Immunofluorescence assays confirmed that all four proteins are localized to the cytosol in transgenic cells (Fig. 7A). Functional analysis revealed that overexpression of CS and TrxR in B7268 significantly reduced MTZ resistance, decreasing the minimum lethal concentration (MLC) from 350 μg/ml to 116.7 μg/ml (p < 0.0001). Overexpression of Myb5 also reduced resistance, with an MLC reduction to 216.7 μg/ml (p = 0.0083). Conversely, overexpression of Myb6 had no significant effect, as the resistance level remained similar to that of the control strain transfected with the empty vector (EpNEO) (Fig. 7B).

These findings confirm the functional roles of TrxR, CS, and Myb5 in modulating MTZ resistance and suggest that their epigenetic silencing in the resistant strain B7268 is a key mechanism underlying the development of drug resistance.

## DISCUSSION

Metronidazole (MTZ) resistance in *T. vaginalis* is a complex and multifactorial process, with several mechanisms contributing to its development. Clinical resistance has been indirectly linked to reduced activity of oxygen scavenging enzymes, such as flavin reductase and thioredoxin reductase (TrxR), which can elevate intracellular oxygen levels and impair MTZ activation [10, 12]. While this mechanism is well-documented *in vitro* in strains with aerobic resistance [11], clinical resistance more frequently involves mutations or enzymatic dysfunctions in hydrogenosomal activation pathways, such as ferredoxin and nitroreductases [15, 16]. Additionally, MTZ resistance has been associated with single nucleotide polymorphisms (SNPs) across 72 genes [15], including some functionally characterized drug resistance genes like pyruvate:ferredoxin oxidoreductase [15] or nitroreductase genes [16].

Now, our study demonstrates for the first time that epigenetic regulation also contribute to modulate MTZ resistance in *T. vaginalis*. Using trichostatin A (TSA), an inhibitor of histone deacetylase (HDAC), we observed significant genome-wide transcriptional changes and increased MTZ sensitivity in a highly resistant strain. These findings align with previous studies highlighting the role of epigenetic regulators, such as DNA methylation and histone acetylation, in modulating gene expression in *T. vaginalis* [31, 52, 53]. Similar effects have been observed with Resveratrol, a natural polyphenolic compound known to elicit diverse epigenetic modifications such as alterations in DNA methyltransferases, HDACs, and lysine-specific demethylase [54]. This compound has previously demonstrated its ability to induce antiparasitic activity through the modulation of gene expression and hydrogenosomal dysfunction in *T. vaginalis* [55]. Similarly, there is evidence that chromatin structure plays a vital role in the acquisition and maintenance of anti-fungal resistance in *Candida spp*. Various chromatin modifiers have directly implicated in the ability of *Candida spp*. to survive in the presence of anti-fungal drugs [56]. For instance, the deletion of the Rpd3/Hda1 family of histone deacetylases in *C. albicans* sensitizes the yeast to fluconazole, itraconazole, and voriconazole [57]. Consistently, the removal of the Histone Acetyltransferase Hat1 achieves the reverse, increasing *C. albican*’s azole resistance [58]. These parallels suggest that chromatin modifiers are critical in drug resistance mechanisms across various pathogens and highlight the promising role of HDACs as novel drug targets for combating parasitic infections.

Considering that TSA treatment reduces drug resistance and that increased histone acetylation generally enhances gene expression, we propose that certain genes may be epigenetically silenced in the resistant strain. Specifically, treatment of a highly resistant T. vaginalis strain (B7268) with TSA or TSA+MTZ led to the upregulation of 1,464 genes, including those involved in transcriptional regulation, oxidoreductase activity, and redox homeostasis. Among these, 130 candidate genes were also differentially expressed in the sensitive strain NYH209. We hypothesize that some of these genes, epigenetically silenced in the resistant B7268 strain, play a role in modulating MTZ resistance. Supporting this hypothesis, overexpression of three candidate genes—cysteine synthase (CS), thioredoxin reductase (TrxR), and Myb5—in the resistant strain partially restored MTZ susceptibility, demonstrating their involvement in resistance modulation. Redox balance plays a pivotal role in MTZ activation. A clear connection between metronidazole resistance and redox enzymes has been previously reported for *E. histolytica* and *T. vaginalis* [59]. Indeed, total insensitivity to metronidazole can be rapidly induced in *T. vaginalis* by treatment with diphenylene iodonium (DPI), which inhibits all flavoenzymes in the parasite, including TrxR, thereby blocking the activation of MTZ [60]. An evident candidate enzyme in this regard is TrxR, functioning as a central redox regulator governing numerous processes, with a pivotal role in the reduction of peroxiredoxin via thioredoxin [59]. Also, TrxR was proposed as a target of metronidazole in *T. vaginalis* as 5-nitroimidazoles can form covalent adducts with cysteines in TrxR and inhibit the enzyme’s activity [10]. Similarly, nitroreductases (ntr4) have presented mutations associated with the development of resistance in *T. vaginalis* parasites [16, 61]. In addition, the redox equilibrium in the cell is not only shaped by redox enzymes but also by low molecular mass thiols and cysteine, synthesized by cysteine synthase, that constitute the main cellular redox buffers in *T. vaginalis* [59, 62]. Resistant strains often exhibit metabolic trade-offs, one potential adaptive mechanism involves the downregulation of cysteine synthase, which may reduce the levels of intracellular cysteine, the precursor for thiol-based molecules critical for detoxifying reactive oxygen species (ROS). As the decrease in cysteine and thiol availability could avoid the formation of covalent adducts with activated MTZ, it also comes at the cost of impaired thiol-based ROS neutralization, forcing resistant strains to rely on alternative antioxidant systems such as SOD or peroxiredoxins [13, 63–65]. Thus, the downregulation of cysteine synthase represents a striking example of how resistant *T. vaginalis* strains may exploit a delicate balance between reducing thiols to evade drug toxicity and maintaining sufficient oxidative stress defense. These findings lay the groundwork for further research into the role of specific *T. vaginalis* genes in the development of drug resistance, including novel candidates such as Myb5, a Myb-like DNA-binding domain-containing protein, whose contribution to resistance development has not been suggested previously in *T. vaginalis*. In this sense, the role of Myb proteins has been proposed in cancer drug resistance [66]. Future studies should focus on elucidating the mechanisms by which these genes influence drug resistance and explore whether the simultaneous reconstitution of multiple silenced genes could further enhance MTZ susceptibility.

Our findings suggest that epigenetically silenced genes significantly contribute to MTZ resistance in *T. vaginalis*. A deeper understanding of these newly identified mechanisms will provide better insights into the development of novel drug targets and potentially lead to more efficient treatment options. Targeting HDAC enzymes as parasitic drug therapies is a promising strategy. Evidence from *Plasmodium falciparum* and *Trypanosoma brucei* shows that resistance mechanisms maintained through a coordinated interplay of acetylation, methylation, and chromatin remodeling processes rather than relying on a single epigenetic mechanism [33, 34, 36, 38]. These studies also lay the foundation for further examinations of newly identified genes and pathways that can be investigated to formulate new treatments, particularly for patients who have developed MTZ resistance.

## Supplementary Material

Supplement 1

Supplement 2

Supplement 3

Supplement 4

1

## Figures and Tables

**Table 1:** 

	Total number of genes	TSA & TSA_MTZ	TSA_MTZ
Unspecified product	301	224	77
Ubiquitin related processes and proteasome system	204	162	42
Kinase/phosphatase activity	117	97	20
Ankyrin repeat containing proteins	107	89	18
Transcription regulation	78	77	1
Genomic modifiers (transposases and endonucleases)	76	60	16
Pathogenic related proteins	58	52	6
Ribonuclease activity and regulation	25	20	5
Transporters	25	15	10
Cell redox balance	18	15	3
Structural proteins	12	10	2
RNA processing and binding	11	10	1
Chromatin structure regulation	8	4	4
