# Supplementary figures and images for: Targeting histone acetylation to overcome drug resistance in the parasite Trichomonas vaginalis
Source: bioRxiv. 2025 Jan 7:2025.01.07.631743. Preprint. [Version 1] doi: 10.1101/2025.01.07.631743 (PMC11741363; doi:10.1101/2025.01.07.631743)

Fig S1

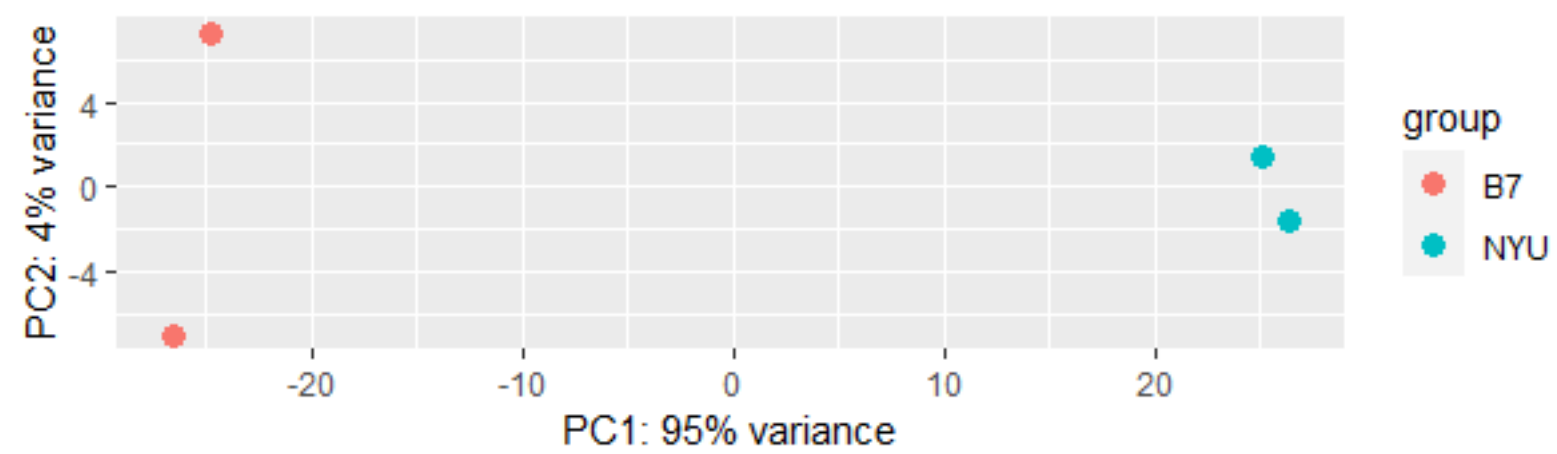

Supplement: 1 [file NIHPP2025.01.07.631743V1-supplement-1.pdf]
